# Synthesis, Characterization, and Retinol Stabilization of Fatty Amide-β-cyclodextrin Conjugates

**DOI:** 10.3390/molecules21070963

**Published:** 2016-07-22

**Authors:** Hwanhee Kim, Yiluo Hu, Daham Jeong, Bong-Hyun Jun, Eunae Cho, Seunho Jung

**Affiliations:** 1 Department of Bioscience and Biotechnology, Microbial Carbohydrate Resource Bank (MCRB), Konkuk University, 120 Neungdong-ro, Gwangjin-gu, Seoul 05029, Korea; hwanhee@konkuk.ac.kr (H.K.); lannyhu0806@hotmail.com (Y.H.); amir@konkuk.ac.kr (D.J.); bjun@konkuk.ac.kr (B.-H.J.); 2 Center for Biotechnology Research in UBITA (CBRU), Institute for Ubiquitous Information Technology and Applications (UBITA), Konkuk University, 120 Neungdong-ro, Gwangjin-gu, Seoul 05029, Korea

**Keywords:** amphiphilic cyclodextrin, self-assembly, all-*trans*-retinol, stabilization

## Abstract

Amphiphilic cyclodextrin (CD) has been the object of growing scientific attention because of its two recognition sites, the cavity and the apolar heart, formed by self-assembly. In the present study, mono[6-deoxy-6-(octadecanamido)]-β-CD and mono[6-deoxy-6-(octadecenamido)]-β-CD were successfully synthesized by reacting mono-6-amino-6-deoxy-β-CD with *N*-hydroxysuccinimide esters of corresponding fatty acids in DMF. The structures were analyzed using nuclear magnetic resonance spectroscopy and mass spectrometry. The amphiphilic β-CDs were able to form self-assembled nano-vesicles in water, and the supramolecular architectures were characterized using fluorescence spectroscopy, dynamic light scattering, and transmission electron microscopy. Using the cavity-type nano-vesicles, all-*trans*-retinol was efficiently encapsulated; it was then stabilized against the photo-degradation. Therefore, the present fatty amide-β-CD conjugate will be a potential molecule for carrier systems in cosmetic and pharmaceutical applications.

## 1. Introduction

Cyclodextrins (CDs) are enzymatic degradation products from starch bioconversion, consisting of six, seven, and eight glucopyranose units linked by α-(1→4) bonds; they are known as α-, β-, and γ-CDs, respectively [1]. Since CDs have torus-like structures incorporating hydrophobic guest compounds, they can solubilize poorly-soluble drugs and protect active ingredients from chemical degradation [2]. Due to its abundance, β-CD has generally been used in enzyme mimetics or drug delivery systems [3,4]. Furthermore, numerous chemically modified CDs have been synthesized to enhance and specify functionality [5].

Amphiphilic CDs represent a new generation of modified CDs; interestingly, they are capable of forming micellar aggregates or vesicles [6,7,8,9]. By hydrophobic modification, the possibility of intimate contact of CDs with biological membranes can also be improved [10]. Based on these properties, amphiphilic CDs can be used in surface activity and solubilization, as well as in drug delivery. The amphiphilic CDs are classified as medusa-like, skirt-shaped, bouquet-shaped, ladle-type, and lollipop-style CDs according to the substitution sites and numbers [11,12]. In particular, lollipop style CDs with mono-substitution on the primary side are designed to enhance cell targeting of drug-CDs, leaving the secondary face open and accessible to guest compounds [13]. 

Vitamin A (all-*trans*-retinol), as a fat-soluble vitamin, is essential for immune reactions, embryonic growth, vision, reproduction, and epidermal differentiation [14]. Thus, it has attracted interest for nutritional, pharmaceutical, and cosmetic applications. Furthermore, since all-*trans*-retinol cannot be synthesized in humans, it has to be supplemented by dietary sources, such as milk fat, egg yolks, and mammalian liver [15]. For the large scale processes, biotechnological production has also been carried out [16]. However, the structure with *trans* double bonds in the isoprenoid side chain is liable to decompose under light and oxygen [17]. Degradative reactions can cause undesired side effects including aging, inflammation, and cardiovascular diseases as well as loss of retinol bio-functionality [18]. Therefore, formulation strategies to enhance vitamin A stability are highly required; previous efforts have focused on the incorporation of vitamin A into CDs [19], liposomes [20], microspheres [21], and nanoparticles [22]. In addition, retinyl palmitates are often used as a synthetic alternative for vitamin A supplements [23].

Herein, we have aimed at combining and conjugating a fatty acid and the β-CD moiety for the efficient encapsulation and protection of all-*trans*-retinol. For a saturated and unsaturated fatty acid model, stearic acid (C18:0) and oleic acid (C18:1:Δ^9^) were chosen, and mono-substitution on the primary side of β-CD was carried out. The resulting lollipop-style β-CDs (mono[6-deoxy-6-(octadecan amido)]-β-CD and mono[6-deoxy-6-(octadecenamido)]-β-CD) form supramolecular architectures in water, which are analyzed with fluorescence spectroscopy, dynamic light scattering (DLS), and transmission electron microscopy (TEM). Compared with free all-*trans*-retinol, the present stearamido-β-CD hybrid molecule which forms nano-vesicles shows marked efficiency in all-*trans*-retinol loading and stabilization.

## 2. Results and Discussion

### 2.1. Structural Analysis of Mono[6-Deoxy-6-(Octadecanamido)]-β-CD (Stearamido-β-CD, S-β-CD) and Mono[6-Deoxy-6-(Octadecenamido)]-β-CD (Oleamido-β-CD, O-β-CD)

**Scheme 1:**
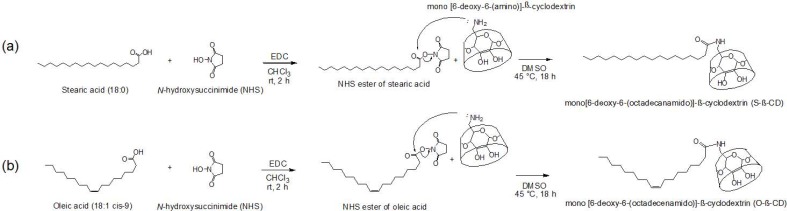
Synthetic schemes. (**a**) S-β-CD; and (**b**) O-β-CD.

**Figure 1:**
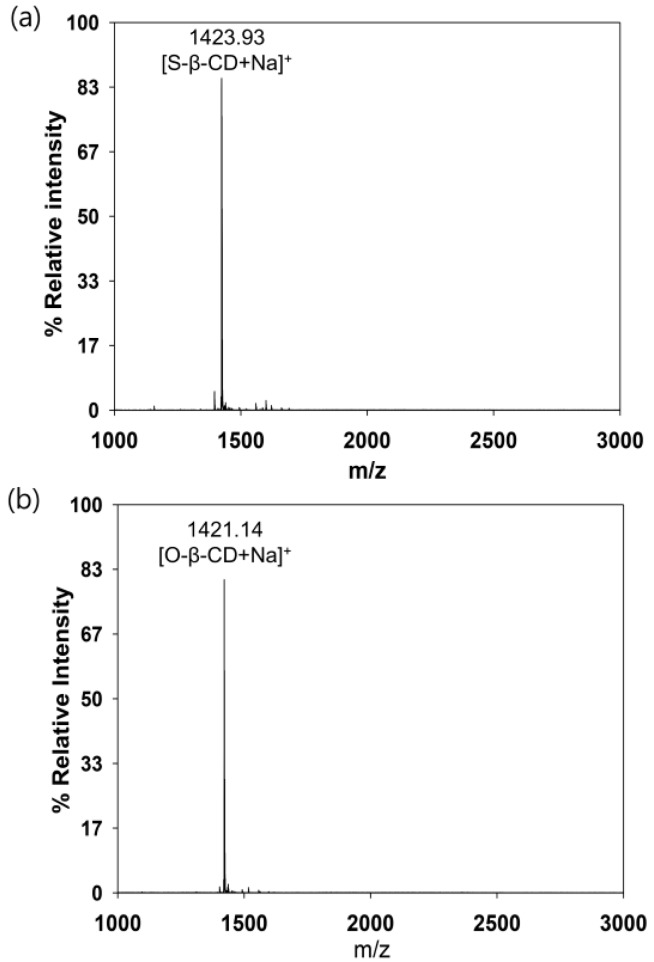
MALDI TOF mass spectra. (**a**) S-β-CD; and (**b**) O-β-CD.

**Figure 2:**
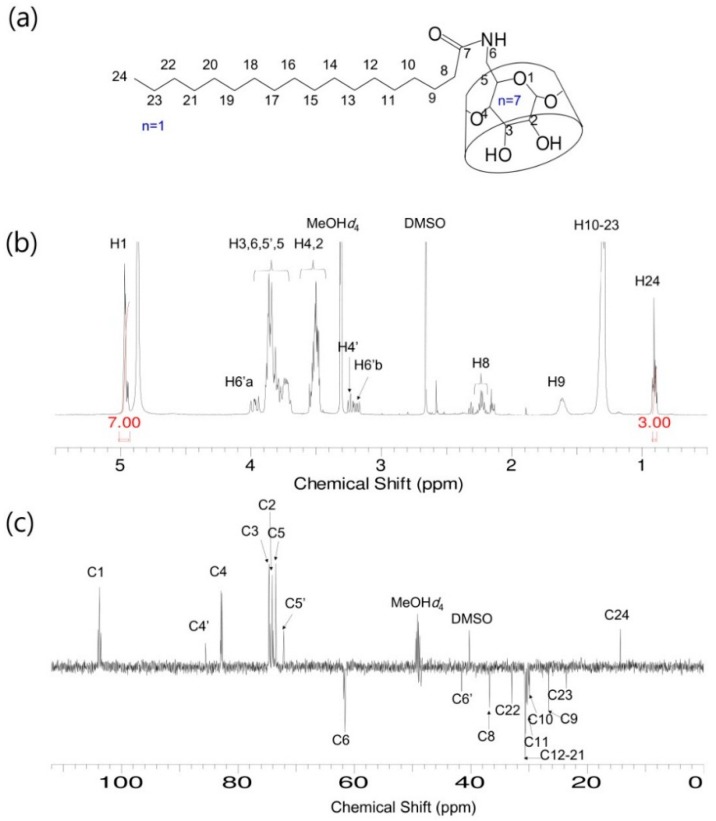
S-β-CD. (**a**) Chemical structure; (**b**) ^1^H-NMR spectrum; and (**c**) DEPT-135 NMR spectrum. Solvent: MeOH-*d*_4_.

**Figure 3:**
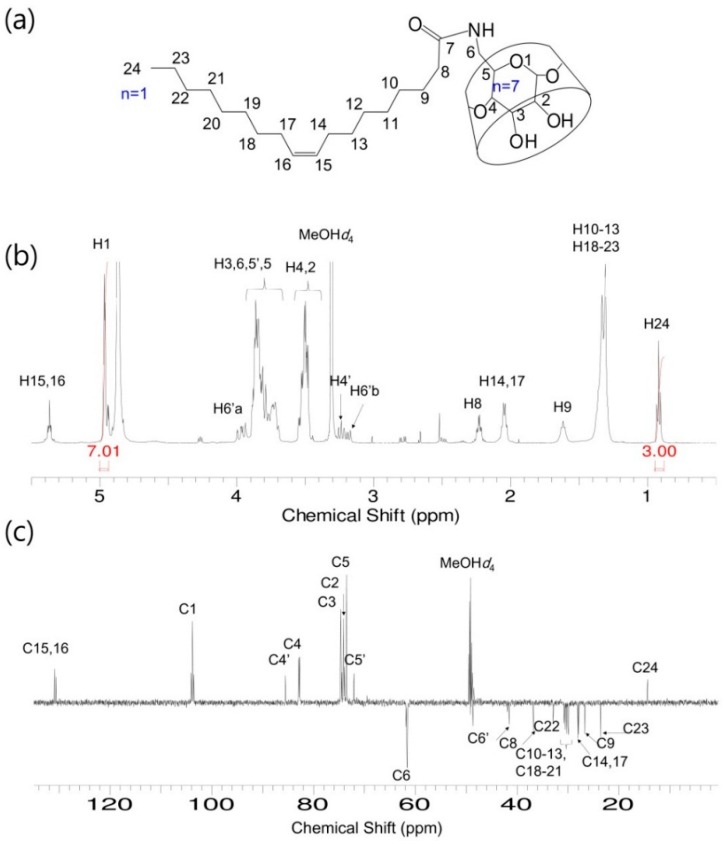
O-β-CD. (**a**) Chemical structure; (**b**) ^1^H-NMR spectrum; and (**c**) DEPT-135 NMR spectrum. Solvent: MeOH-*d*_4_.

S-β-CD and O-β-CD were synthesized as described in the experimental section (Scheme 1). After separation from the reaction mixtures, the resulting products were analyzed using matrix-assisted laser desorption/ionization-time of flight (MALDI-TOF) mass spectrometry and nuclear magnetic resonance (NMR) spectroscopy. The pseudo-molecular ion peak at *m*/*z* = 1423.93 was assigned to [S-β-CD + Na]^+^, and the peak at *m*/*z* = 1421.14 of [O-β-CD + Na]^+^ proved the presence of one double bond on the same carbon chain length (C18) (Figure 1). The chemical structure of S-β-CD is illustrated in Figure 2a; the mono-substitution is also confirmed by the integration of H1 and H24 protons (Figure 2b). The substitution at the C6 position induces moderate upfield and downfield shifts of the H4′ and H5′ protons as well as a shift of the H6′ab protons. In DEPT-135 spectrum, it was possible to distinguish methylene carbons for C6 and C8–23 (Figure 2c). In addition, a large upfield chemical shift of the C6′ carbons (δ 41.61), a small upfield shift of C5′ (δ 72.12), and a small downfield shift of C4′ (δ 85.56) were observed; on the other hand, there is no special change in the chemical shift of C1. In the case of O-β-CD (Figure 3a), the protons (H15 and H16) attached to double bonds appear farthest downfield (δ 5.32–5.42) due to the diamagnetic anisotropy generated by the pi electrons, and the *cys* coupling constant is found to be 7.5 Hz (Figure 3b). The carbons attached to the double bond are also deshielded to δ 130.91 and 130.65 due to their sp^2^ hybridization and diamagnetic anisotropy (Figure 3c). Other shifting patterns are similar to those of S-β-CD. These structural analyses indicate successful mono-stearamide or mono-oleamide modification on the C6 of β-CD.

### 2.2. Self-Assembly of S-β-CD and O-β-CD in Water

**Figure 4:**
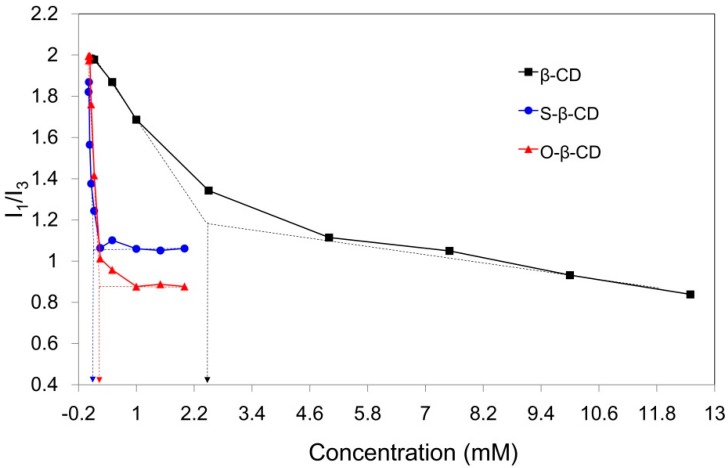
Relationship of pyrene fluorescence intensity ratios (I_1_/I_3_) with the concentration of β-CD (black line), S-β-CD (blue line), and O-β-CD (red line). The arrows indicate the cac values of β-CD, S-β-CD, and O-β-CD.

Compared with original β-CD (18.2 mg/mL, 25 °C), the synthesized S-β-CD and O-β-CD show lower aqueous solubilities (0.6 and 1.6 mg/mL, 25 °C) due to the substituted stearamide and oleamide moieties (Table 1). This result is also confirmed through determination of the critical aggregation concentration (cac) by fluorescence technique. Pyrene, a fluorescent hydrophobic probe, is preferentially solubilized into the interior of lipophilic microenvironments in an aqueous system [24]. From the emission bands of pyrene, the intensity ratio of the first to the third band (I_1_/I_3_) is considered as an indicator of the polarity of the microenvironment around the pyrene moiety. The I_1_/I_3_ values of pyrene in water, methanol, and hexane are 1.87, 1.35, and 0.58, respectively [25]. When the cac is at its lowest concentration the material shows self-aggregation behavior; this can be determined from the intersecting point of the linear extension of the rapidly decreasing part and the horizontal part of the curve (Figure 4). As pyrene experiences the hydrophobic interior of β-CD in water, the I_1_/I_3_ values begin to decrease, and the calculated cac is 2.47 mM. In particular, the stearamide modification on β-CD gives rise to a sharply declining curve and reduced cac (0.09 mM). This decline is attributed to the two hydrophobic microdomains of the β-CD cavity and the self-assembled apolar part around pyrene [12]. The cac value of O-β-CD was determined to be 0.26 mM, slightly higher than that of S-β-CD. The disordered conformation due to the one *cys* double bond on the C18 chain might slightly interrupt the self-aggregation of O-β-CD. These results indicate that self-assembly of S-β-CD and O-β-CD can be used as a carrying system for the desired hydrophobic compounds.

### 2.3. Supramolecular Nano-Vesicles 

**Figure 5:**
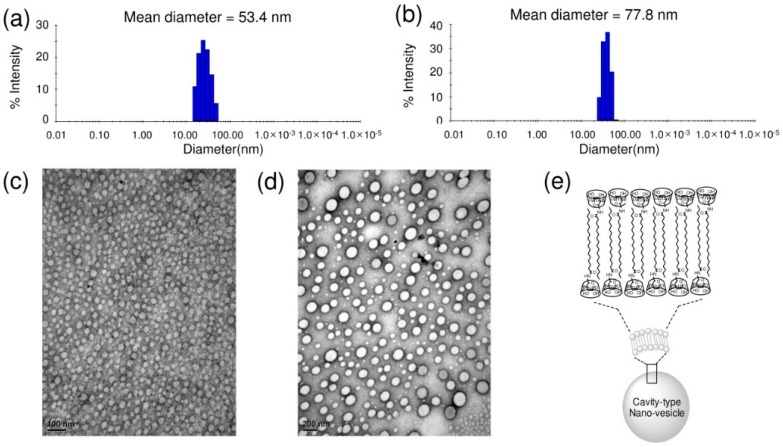
DLS profiles. (**a**) S-β-CD; (**b**) O-β-CD. TEM images; (**c**) S-β-CD (scale bar = 100 nm); (**d**) O-β-CD (scale bar = 200 nm); and (**e**) illustration of the self-assembled nano-structure by S-β-CD in water.

To investigate the size and morphology of the self-assembled architecture, DLS and TEM data are analyzed [26]. Figure 5 shows the mean hydrodynamic diameter and spherical morphology of the nano-aggregates formed in aqueous solution. The self-assembled composites of S-β-CD show an average diameter of 53.4 nm in the DLS profile (Figure 5a). On the other hand, O-β-CD has a larger mean diameter of 77.8 nm in Figure 5b; this may be accounted for by the presence of one double bond. Similar patterns were observed in TEM images; the mean diameters of self-assemblies by S-β-CD and O-β-CD are 24.1 nm and 42.5 nm, respectively (Figure 5c,d and Figure S2a,b in Supplementary Materials). The size differences in DLS and TEM data are possible, since TEM shows the image from the solid state in vacuo as a number-based particle size measurement [27]. Whereas, DLS provides the hydrodynamic diameter of swollen vesicles as a scattering intensity-based particle size measurement [28]. The heterogenous-sized vesicles in Figure 5d can also be attributed to the oleamide structure. Saturated chains will be more favorable for forming the regular and stable assemblies [29]. For mono-substitution, the interdigitated packing of alkyl chains is proposed, as illustrated in Figure 5e [30]. Different building blocks of S-β-CD and O-β-CD could lead to distinctive self-organized nanostructures.

### 2.4. All-Trans-Retinol Encapsulation

**Figure 6:**
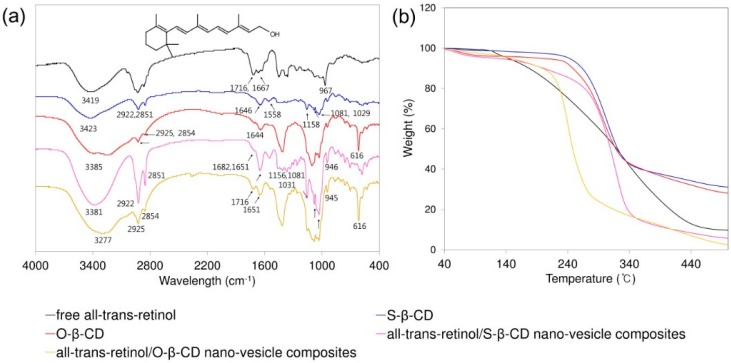
(**a**) FT-IR spectra (Inset shows the chemical structure of all-*trans*-retinol.); and (**b**) TGA curves for the free all-*trans*-retinol, S-β-CD, O-β-CD, all-*trans*-retinol/S-β-CD nano-vesicle composites, and all-*trans*-retinol/O-β-CD nano-vesicle composites.

These cavity-type nano-vesicles made of S-β-CD and O-β-CD are applied to incorporate the lipophilic model compound, all-*trans*-retinol. After incorporation all-*trans*-retinol into the nano-vesicles, the composites were analyzed using FT-IR spectroscopy (Figure 6). The characteristic peaks of all-*trans*-retinol are shown as O–H stretch (3419 cm^−1^), C–H stretch (2927 cm^−1^), C=C stretch (1716 and 1667 cm^−1^), CH_2_, CH_3_ bend (1451 and 1359 cm^−1^), and *trans* out-of-plane bend (967 cm^−1^). S-β-CD has the O–H stretch (3423 cm^−1^), C–H stretch (2922 and 2851 cm^−1^), C=O stretch (1646 cm^−1^), and C–O–C and C–O–H stretch (1158, 1081, 1029 cm^−1^). The spectrum of O-β-CD shows O–H stretch (3224 cm^−1^), C–H stretch (2925 and 2854 cm^−1^), *cis* C=C stretch (1644 cm^−1^), and *cis* out-of-plane bend (616 cm^−1^). In the all-*trans*-retinol/nano-vesicle composites, the peaks of retinol and the building block (S-β-CD or O-β-CD) co-exist, and the observed peak shifts are considered for interactions between the compounds [31]. In addition, the thermal stabilities were assessed by TGA [32]. Although relative stability is higher in S-β-CD nano-vesicle composites than in O-β-CD nano-vesicle composites, both composites make the degradation temperature lower than the respective building blocks due to the all-*trans*-retinol. The results indicate that all-*trans*-retinol could be successfully encapsulated into nano-vesicles.

Furthermore, the encapsulation efficiency was investigated; it was calculated according to the proportion of the amount of all-*trans*-retinol in the nano-vesicles to the total amount of all-*trans*-retinol [33]. The encapsulation efficiency of S-β-CD (50.18%) was better than that of O-β-CD (21.68%) (Table 2). Since all-*trans*-retinol can be incorporated into the β-CD cavity or among the hydrophobic tails, the nano-vesicles well-organized by S-β-CD may have a beneficial effect on the effective encapsulation.

### 2.5. All-Trans-Retinol Stabilization

**Figure 7:**
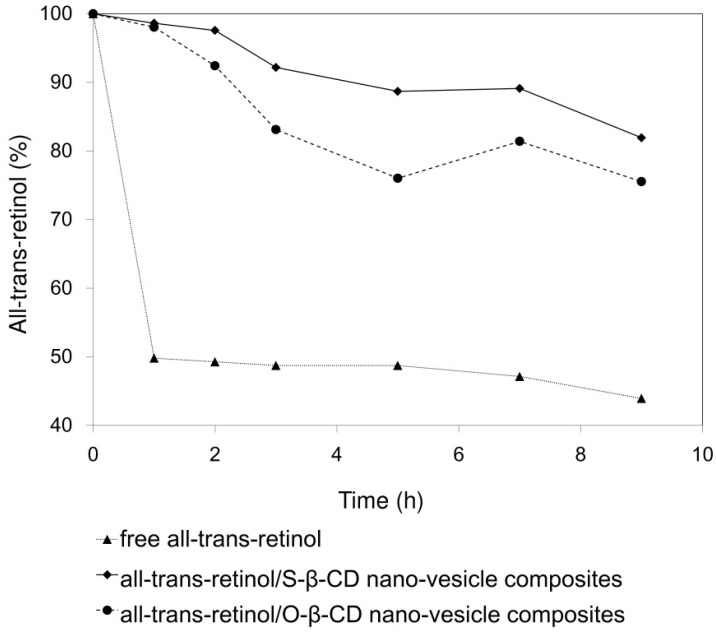
Photo-degradation curves, under UVA irradiation, of all-*trans*-retinol, all-*trans*-retinol/S-β-CD nano-vesicle composites, and all-*trans*-retinol/O-β-CD nano-vesicle composites in aqueous solution.

Since all-*trans*-retinol is a photo-sensitive and labile substance, stability increase is an important factor for its formulation and efficacy. Residual percentages of all-*trans*-retinol after UV exposure are plotted for the control, S-β-CD nano-vesicle, and O-β-CD nano-vesicle samples (Figure 7). In water (control), 50% of free all-*trans*-retinol decomposed within 1 h. On the other hand, all-*trans*-retinol encapsulated within S-β-CD nano-vesicle maintained 82% of its initial content even after 9 h UV exposure. In the case of the O-β-CD system, relatively reduced stability was observed with 76% residual all-*trans*-retinol. In general, comparing with saturated fatty acids, unsaturated fatty acids result in more flexible tails, which destabilize the lamellar bilayer [34]. Considering that, O-β-CD having one cis double bond might be unfavorable to form nano-vesicles via interdigitated packing as depicted in Figure 5e. Thus, based on its regular and organized cavity-type nanovesicular structure, S-β-CD could be more effective in the encapsulation and photo-stability of all-trans-retinol than O-β-CD.

## 3. Materials and Methods

### 3.1. Chemicals

β-CD (97%), oleic acid, and DMSO were obtained from Tokyo Chemical Industry Co., Ltd. (Tokyo, Japan). All-*trans*-retinol and stearic acid were purchased from Sigma-Aldrich Chemicals Co. (Sigma-Aldrich, St. Louis, MO, USA). NHS was obtained from Fluka (Fluka Chemie AG, Buchs, Switzerland). 1-(3-Dimethylaminopropyl)-3-ethyl carbodiimide hydrochloride (EDC) was purchased from Acros Organics (Morris, NJ, USA). Organic solvents, such as chloroform, hexane, ethyl acetate, acetone, and *n*-propyl alcohol, were of analytical grade, and the high-purity (Milli-Q, Darmstadt, Germany) water was used through the study.

### 3.2. Synthesis

#### 3.2.1. *N*-Hydroxysuccinimide (NHS) Esters of Stearic Acid and Oleic Acid

NHS esters of fatty acids were synthesized based on the previous report [35]. Briefly, stearic acid and oleic acid (1.53 g, 5.4 mmol) were dissolved in 20 mL of chloroform. NHS (675 mg, 5.8 mmol), and EDC (1.23 g, 7.9 mmol) were added to the organic solvent. The resulting solution was stirred at room temperature for 4 h. After removing the solvent in vacuo, the product was extracted with ethyl acetate and washed with water. The organic layer was dried with magnesium sulfate and filtered; the solvent was evaporated by rotary evaporation. The product was then recrystallized and a clean product was obtained at 80% yield. The product was confirmed using thin-layer chromatography (TLC, hexane/ethyl acetate 3:1).

#### 3.2.2. Synthesis of S-β-CD and O-β-CD

Mono-6-amino-6-deoxy-β-CD was prepared from the mono-6-*O*-*p*-toluenesulfonyl-β-CD [36]. Mono-6-amino-6-deoxy-β-CD (50 mg, 45 μmol) was added to NHS esters of stearic acid and oleic acid (36 mg, 90 μmol) in 1 mL of DMSO. The mixture was stirred for 18 h at 45 °C and then purified using flash chromatography (*n*-propyl alcohol: methanol: ethyl acetate = 7:3:3). The chemical structures of the fatty amido-β-CDs were determined by MALDI-TOF spectrometry and NMR spectroscopy.

*S-β-CD*. ^1^H-NMR (500MHz, MeOH-*d*_4_): δ 4.97–4.94 (m, 7H, H1), 4.00–3.94 (m, 1H, H6′a), 3.89–3.69 (m, H3,6,5′,5), 3.55–3.47 (m, H4,2), 3.26–3.22 (m,1H, H4′), 3.21–3.17 (m, 1H, H6′b), 2.28–2.18 (m, 2H, H8), 1.65–1.55 (m, 2H, H9), 1.36–1.24 (m, 28H, H10–23), 0.92–0.89 (m, 3H, H24); ^13^C-NMR (500 MHz, MeOH-*d*_4_): δ 103.79 (C1), 85.56 (C4′) 82.92 (C4), 74.67 (C3), 74.12 (C2), 73.52 (C5), 72.12 (C5′), 61.63 (C6), 41.61 (C6′), 36.80 (C8), 32.96 (C22), 30.66 (C12–21), 30.31 (C11), 29.94 (C10), 26.60 (C9), 23.61(C23), 14.35 (C24); MALDI-TOF MS: 1423.93 [S-β-CD + Na]^+^.

*O-β-CD*. ^1^H-NMR (500MHz, MeOH-*d*_4_): δ 5.38–5.36 (m, 2H, H15,16), 4.97–4.94 (m, 7H, H1), 4.00–3.94 (m, 1H, H6′a), 3.86–3.70 (m, H3,6,5′,5), 3.54–3.47 (m, H4,2), 3.26–3.22 (m, 1H, H4′), 3.21–3.17 (m, 1H, H6′b), 2.25–2.21 (m, 2H, H8), 2.07–2.03 (m, 2H, H14,17), 1.63–1.60 (m, 2H, H9), 1.38–1.31 (m, 20H, H10–13, H18–23), 0.93–0.90 (m, 3H, H24); ^13^C-NMR (500 MHz, MeOH-*d*_4_): δ 130.91 (C15), 130.65 (C16), 103.82 (C1), 85.55 (C4′), 82.89 (C4), 74.66 (C3), 74.14 (C2), 73.52 (C5), 72.06(C5′), 61.64 (C6), 41.60 (C6′), 36.83 (C8), 32.94 (C22), 30.81–29.88 (C10–13, C18–21), 28.10 (C14,17), 26.70 (C9), 23.61 (C23), 14.40 (C24); MALDI-TOF MS: 1421.14 [O-β-CD + Na]^+^.

### 3.3. Characterization

#### 3.3.1. MALDI-TOF Mass Spectrometry

The mass spectrum was obtained using a MALDI-TOF mass spectrometer (Voyager-DETM STR BioSpectrometry, PerSeptive Biosystems, Framingham, MA, USA) using the positive-ion mode. 2, 5-Dihydroxybenzoic acid (DHB) was used as the matrix.

#### 3.3.2. NMR Spectroscopy

For the NMR spectroscopic analysis, a Bruker Avance 500 spectrometer (Bruker, Karlsruhe, Germany) was used to record the ^1^H-NMR, DEPT-135 and HSQC spectra. The HSQC spectrum was measured with a spectral width of 6355 Hz in both dimensions and 256/2048 complex data points in t1 and t2, respectively. NMR analyses were performed in MeOH-*d*_4_ at room temperature. 

### 3.4. Measurement of Fluorescence Spectroscopy

The cac value was determined by fluorescence probe, as previously described by Liu et al. [37]. In brief, 1 μL of pyrene solution was aliquoted into a series of 5 mL vials. A series of aqueous CD solutions (concentration from 0.008125 to 12.5 mg/mL) were added separately to obtain the final concentration of pyrene of 1 µM; solutions were then sonicated for 15 min at room temperature, and finally incubated at 25 °C for 16 h to ensure pyrene incorporation into the vesicles. After the samples were allowed to sit overnight, pyrene emission spectra were obtained using a Shimadzu RF-5310PC fluorescence spectrophotometer (Shimadzu Co., Kyoto, Japan). For measurements of the intensity ratios for the first and third peaks (I_1_/I_3_) in the emission spectra for pyrene, the slit openings for excitation and emission were set at 3 and 1.5 nm, respectively. The fluorescence intensity was calculated and plotted as a function of the CD derivative concentrations. Using linear regression, equations describing the two linear parts of the plot were established. The cac was then obtained from the intersection of these two lines. 

### 3.5. Aqueous Solubility of Fatty Amide-β-CD Conjugates

A suspension of fatty amide-β-CD conjugates (5 mg) in 1 mL of H_2_O was sonicated in an Ultrasonic Cleaner (Saehan Ultrasonic Co., Seoul, Korea) for 30 min and then stirred during incubation (25 °C) overnight. When complete dissolution of the fatty amido-β-CDs had not occurred, the mixture was centrifuged (CF-10, Wise Spin, Seoul, Korea); supernatants were then lyophilized.

### 3.6. Characterization of Nano-Vesicles

#### 3.6.1. DLS

DLS measurements were carried out with a DynaPro Plate Reader (Wyatt Technology Corporation, Santa Barbara, CA, USA) at constant room temperature.

#### 3.6.2. TEM

A drop of nanoparticles suspension was placed onto a Formvar-coated copper grid (200 mesh) and air-dried. For negative staining, 2% uranyl acetate solution was used. Observation was performed at 80 kV using a transmission electron microscope (JEOL, JEM 1010, Tokyo, Japan).

### 3.7. Preparation of All-Trans-Retinol Encapsulated Nano-Vesicles

All-*trans*-retinol encapsulated nano-vesicles were prepared by nanoprecipitation method. S-β-CD and O-β-CD were added to ethanol at concentrations of 3 μmol and sonicated for 2 h. To these solutions, all-*trans*-retinol solutions (1.3 μmol) dissolved in ethanol were added. To obtain nano-vesicles loaded with all-*trans*-retinol, the suspensions were added under mechanical stirring to the aqueous phase without surfactant in a drop-wise manner. The nano-vesicles formed immediately and the mixture solution was stirred during incubation at 25 °C overnight. Then, ethanol was evaporated using nitrogen (N_2_) gas. The suspensions were lyophilized to totally remove the ethanol. After lyophilizing, deionized water was added to the lyophilizing samples and the suspension was centrifuged. After removing the all-*trans*-retinol precipitate from outside of the nano-vesicles, the supernatant containing all-*trans*-retinol loaded nano-vesicles was collected.

### 3.8. Fourier-Transform Infrared (FT-IR) Spectroscopy

The all-*trans*-retinol/S-β-CD or O-β-CD nano-vesicle composites are prepared as described in Section 3.7. After lyophilizing, the FT-IR spectra were obtained in a KBr matrix using Nicolet iS50 FT-IR (Thermo Fisher Scientific, Waltham, MA, USA). The spectra were recorded in the scanning range of 400–4000 cm^−1^.

### 3.9. Thermogravimetric Analysis (TGA)

The all-*trans*-retinol/S-β-CD or O-β-CD nano-vesicle composites are prepared as described in Section 3.7. After lyophilizing, TGA was performed on a SDT 2960 simultaneous DTA/TGA analyzer (TA Instruments, Delaware, DE, USA), with a heating rate of 10 °C/min from room temperature to 400 °C under nitrogen gas.

### 3.10. Photostability Study 

The photo-stabilities of the free all-*trans*-retinol and of the nano-vesicles containing 3 μmol S-β-CD and O-β-CD were tested through quantitative analysis using UV-2450 spectrophotometer (Shimadzu Corporation). Samples were prepared as described in section 3.7. The photo-stability of the free and encapsulated all-*trans*-retinol was studied using the output of a UV lamp (H125-BL, 125W, INTERLIGHT, Hammond, IN, USA) with a maximum emission wavelength of ca. 360 nm (UVA radiation). Nano-vesicle solutions were thermostated at 25 °C and photo-irradiated under UVA irradiation for 10 h under magnetic agitation. Samples were placed 20 cm from light source and different aliquots were collected for analysis at different intervals of time. All-*trans*-retinol loading inside the nano-vesicles was assessed by extracting nano-vesicle solution in ethanol and evaluating the UV absorption of the solution at 288 nm using a UV-VIS spectroscopy (Shimadzu Corporation) [32]. The results were expressed as percentages of all-*trans*-retinol remaining. The results were compared with the control (free all-*trans*-retinol).

## 4. Conclusions

In the present study, we synthesized lollipop-style stearamide-β-CD and oleamide-β-CD conjugates by reacting mono-6-amino-6-deoxy-β-CD and NHS esters of saturated and unsaturated C18 fatty acids in DMF. The amphiphiles were self-assembled into distinctive nano-vesicles in water; these nano-vesicles were characterized using fluorescence spectroscopy, DLS, and TEM. The roles of β-CD cavities and the apolar self-assembled domain were proved to result from the incorporation of pyrene, which consisted of four fused benzene rings. For a practical lipophilic model compound, all-*trans*-retinol was loaded in the cavity-type nano-vesicles, which were evaluated as a non-surfactant delivery system that can provide high stability to the labile compound against UV irradiation. In particular, stearamido-β-CD will be a promising building block for effective carriers in the fields of food, pharmaceuticals, and cosmetics. 

## Figures and Tables

**Table 1:** Physicochemical properties of β-CD, S-β-CD, and O-β-CD.

Kinds of β-CD	Molecular Weight (g/mol)	cac (mM)	Solubility in Water at 25 °C (mg/mL)
β-CD	1134	2.47	18.2
S-β-CD	1423	0.09	0.6
O-β-CD	1421	0.26	1.6

**Table 2:** Encapsulation efficiency of S-β-CD and O-β-CD.

	S-β-CD	O-β-CD
Initial all-*trans*-retinol concentration (μmol)	1.3	1.3
Fatty amide-β-CD concentraion (μmol)	3	3
Encapsulation efficiency (%)	50.18 ^a^	21.68 ^a^

^a^ Encapsulation efficiency (%) = the amount of active ingredient in the nanoparticle (μmol)/total amount of active ingredient in whole system (μmol) × 100.

## Supplementary Materials

Supplementary materials can be accessed at: http://www.mdpi.com/1420-3049/21/7/963/s1.
