# Supplementary material for: Synthesis, Characterization, and Retinol Stabilization of Fatty Amide-β-cyclodextrin Conjugates
Source: Molecules. 2016 Jul 22;21(7):963. doi: 10.3390/molecules21070963 (PMC6273423; doi:10.3390/molecules21070963)
Supplement: Supplementary file 1 [file molecules-21-00963-s001.pdf]

Supplementary Materials: Synthesis, Characterization, and Retinol Stabilization of Fatty Amide-β-cyclodextrin Conjugates

Hwanhee Kim, Hu Yiluo, Daham Jeong, Bong-Hyun Jun, Eunae Cho and Seunho Jung

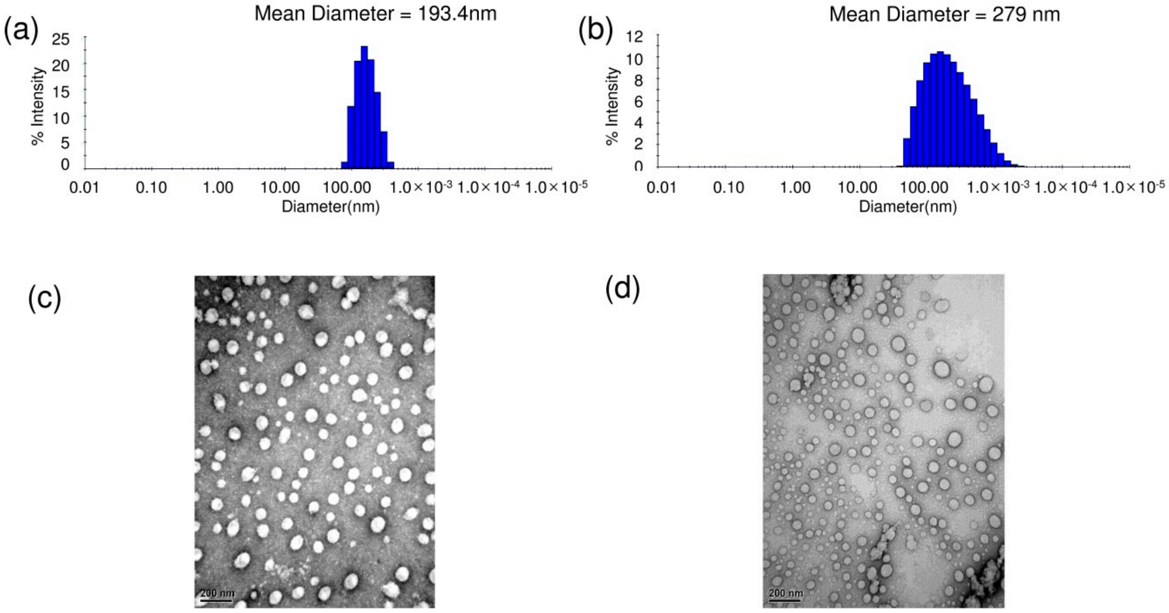

Figure S1. DLS profiles. (**a**) all-*trans*-retinol/S-β-CD nano-vesicles; (**b**) all-*trans*-retinol/O-β-CD nano-vesicles. TEM images. (**c**) all-*trans*-retinol/S-β-CD nano-vesicles (scale bar = 200 nm); (**d**) all-*trans*-retinol/O-β-CD nano-vesicles (scale bar = 200 nm).

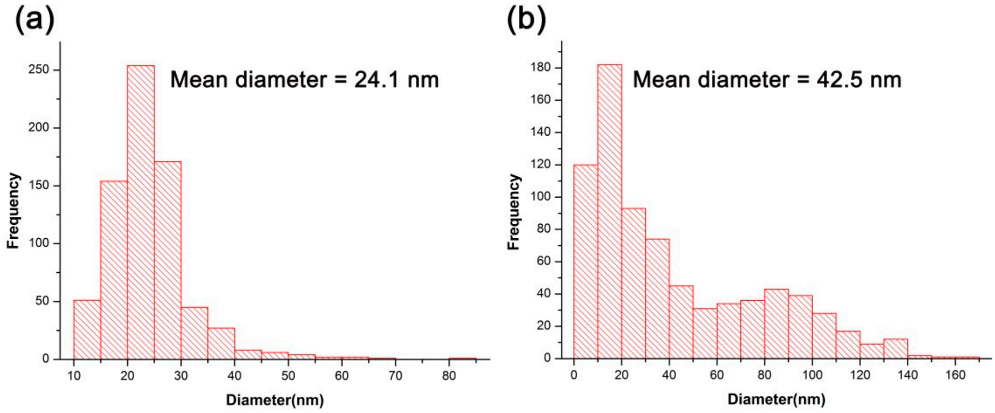

Figure S2. Particle size distributions. (**a**) S-β-CD. (**b**) O-β-CD. For these histograms, a total of 726 (S-β-CD) and 766 (O-β-CD) nano-vesicles from representative TEM images (Figure 5d,e) were analyzed.
